# Optimal treatment for elderly patients with resectable proximal gastric carcinoma: a real world study based on National Cancer Database

**DOI:** 10.1186/s12885-019-6166-3

**Published:** 2019-11-09

**Authors:** Xuefei Wang, Junjie Zhao, Mark Fairweather, Tingsong Yang, Yihong Sun, Jiping Wang

**Affiliations:** 1 Gastric Cancer Center, Department of General Surgery, Zhongshan Hospital, Fudan University, 180 Fenglin Road, Shanghai, 200032 China; 2 Division of Surgical Oncology, Department of Surgery, Brigham and Women’s Hospital, 75 Francis Street, Boston, MA 02115 USA; 3 Department of General Surgery, Shanghai Tenth People’s Hospital, Tongji University, Shanghai, 20072 China

**Keywords:** Proximal gastric carcinoma, Elderly, Surgery, Treatment, National Cancer Database

## Abstract

**Background:**

High perioperative morbidity, mortality, and uncertain outcome of surgery in octogenarians with proximal gastric carcinoma (PGC) pose a dilemma for both patients and physicians. We aim to evaluate the risks and survival benefits of different strategies treated in this group.

**Methods:**

Octogenarians (≥80 years) with resectable proximal gastric carcinoma who were recommended for surgery were identified from National Cancer Database during 2004–2013.

**Results:**

Patients age ≥ 80 years with PGC were less likely to be recommended or eventually undergo surgery compared to younger patients. Patients with surgery had a significantly better survival than those without surgery (5-year OS: 26% vs. 7%, *p* < 0.001), especially in early stage patients. However, additional chemotherapy (HR: 0.94, 95% CI: 0.82–1.08, *P* = 0.36) or radiotherapy (HR: 0.97, 95% CI: 0.84–1.13, *P* = 0.72) had limited benefits. On multivariate analysis, surgery (HR: 0.66, 95% CI: 0.51–0.86, *P* = 0.002) was a significant independent prognostic factor, while extensive surgery had no survival benefit (Combined organ resection: HR: 1.88, 95% CI: 1.22–2.91, *P* = 0.004; number of lymph nodes examined: HR: 0.99, 95% CI: 0.97–1.00, *P* = 0.10). Surgery performed at academic and research (AR) medical center had the best survival outcome (5-year OS: 30% in AR vs. 18–27% in other programs, *P* < 0.001) and lowest risk (30-day mortality: 1.5% in AR vs. 3.6–6.6% in other programs, *P* < 0.001; 90-day mortality: 6.2% in AR vs. 13.6–16.4% in other programs, *P* < 0.001) compared to other facilities.

**Conclusions:**

Less-invasive approach performed at academic and research medical center might be the optimal treatment for elderly patients aged ≥80 yrs. with early stage resectable PGC.

## Background

As the fifth most common malignancy, gastric carcinoma is the third leading cause of cancer deaths in man and fifth in women in the world [1, 2]. Gastric carcinoma is most frequently diagnosed between 65 to 74 years of age [3], with the highest percentage of deaths among people aged 75–84 years [4]. While surgery combined with chemotherapy and/or radiotherapy offers the only curative treatment option, the decision to undergo an aggressive treatment approach for elderly patients is complex [5, 6]. Performance status, comorbidities, and high mortality and morbidity [7, 8], often make both patients and physicians hesitant to pursue radical surgery [9].

Previous studies have reported conflicting outcomes for patients age 80 years and older (≥80 yrs) with gastric carcinoma who undergo surgery [10–13]. A recent study utilizing data from National Surgical Quality Improvement Program (NSQIP) showed that advanced age (≥80 yrs) was associated with major complications and increased mortality [14]. However, studies from Asia have reported that surgery for gastric carcinoma in the elderly has acceptable perioperative morbidity and mortality [15, 16], and have further demonstrated a survival benefit of surgical resection compared to the non-operative management in elderly patients with stage I-III gastric carcinoma [17]. While most carcinomas arise in the distal stomach in Asian countries, nearly 50% of gastric carcinomas arise in the proximal stomach including cardia, fundus and gastroesophageal junction (GEJ) in Western countries [18]. Proximal gastric carcinomas often require an esophagogastrectomy with either an esophagojejunostomy or esophagogastrostomy reconstruction, which are considered to be higher risk procedures associated with higher morbidity and mortality [19–21]. In addition, due to variability of life expectancy, functional reserve of organ systems, social support, and personal preference, the benefit of chemotherapy and radiotherapy remains unclear [22]. As the incidence of proximal gastric carcinoma continues to rise, this is a challenging treatment dilemma that requires urgent attention [11].

Given the underrepresentation of octogenarians in clinical trials, limited evidence has been established to recommend an optimal strategy of treatment for this group of patients. Instead of evaluating the safety and efficacy of surgery between older and younger patient groups [15, 23], our study chose all octogenarians who were considered resectable (stage 0-III, and surgery was recommended by physicians), and aimed to compare the survival outcomes between different treatment strategies for this patients group.

## Methods

### Patient selection

The National Cancer Database (NCDB) is a joint project of the Commission on Cancer of the American College of Surgeons and the American Cancer Society. Based on the International Classification of Diseases for Oncology, Third Revision histology codes (ICD-O-3), patients with gastric carcinoma coded in the range of 8010–8012, 8014–8033, 8042–8148, 8170–8231, and 8252–8576 were eligible for screening in this study. With the approval of the institutional review board, 144,933 patients diagnosed with gastric carcinoma were identified between 2004 and 2013 from the NCDB. Data dictionary Participant User File (PUF) 2014 was used for reference [24]. Charlson-Deyo Comorbidity Index (CDCI) was used to measure the risk of the patients’ comorbidities.

**Fig. 1:**
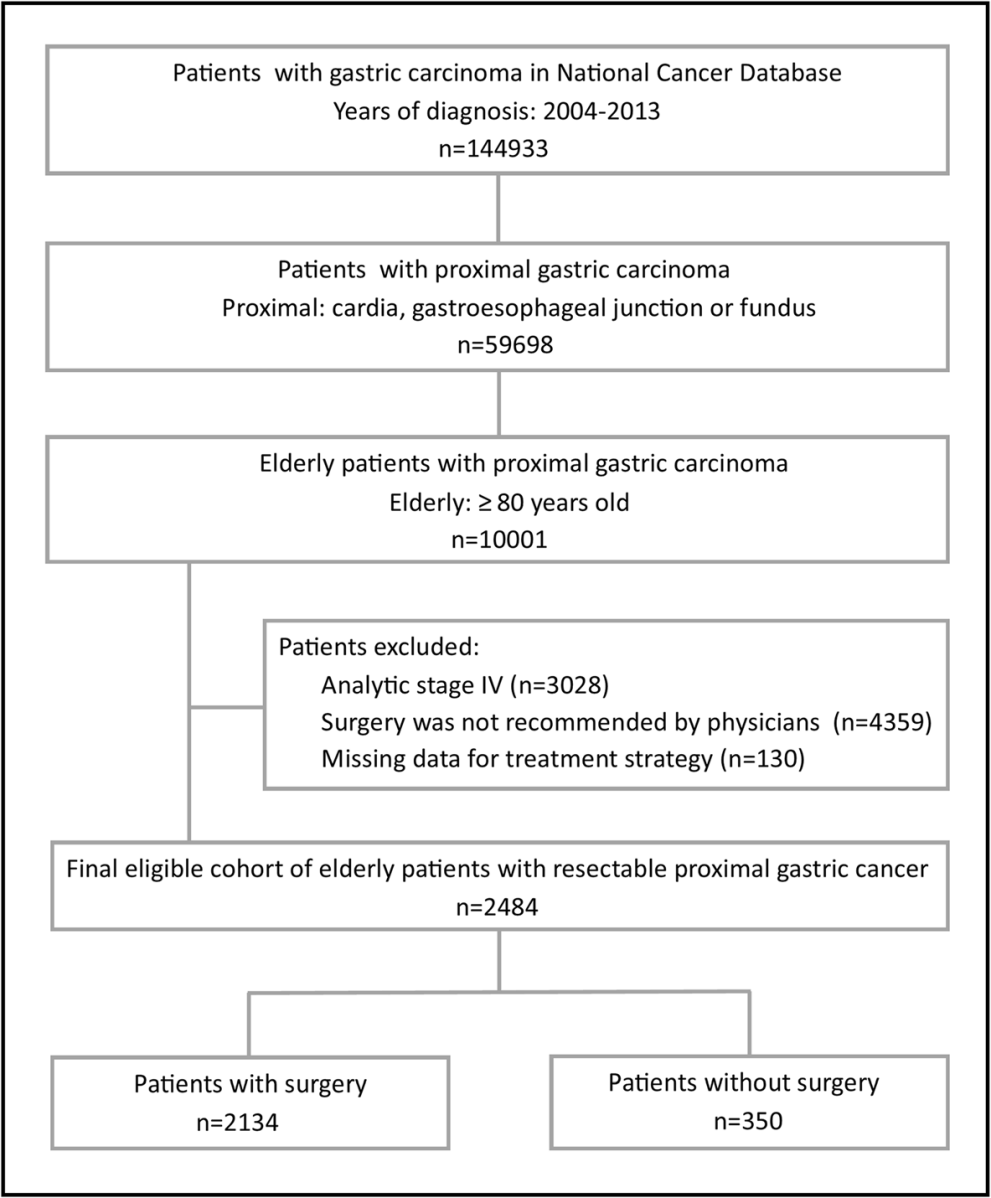
Diagram of cohort selection from National Cancer Data Base

Patients aged ≥80 yrs. with proximal gastric carcinoma were selected according to the site codes of ICD-O-3 with cardia (C16.0), GEJ (C16.0) and fundus (C16.1). The potential reasons for not undergoing a cancer-related surgery were recorded in the NCDB (Surgery was not recommended by physicians or surgery was recommended by physicians but was refused by patient, patient’s family member or guardian, or patient died prior to planned surgery). Patients with stage IV disease, those who were not recommended for surgery (Surgery was not recommended/performed because it was not part of the planned first course treatment or Surgery was not recommended/performed, contraindicated due to patient risk factors) and patients with missing data of treatment strategy were excluded. The stepwise process of data extraction is depicted in Fig. 1.

### Statistical analyses

Baseline characteristics were compared using the Pearson’s χ2 test for categorical variables and student T test for continuous variables (Age is being analyzed as a continuous variable, and interval increment is 1-year). The Kaplan–Meier method was used to estimate overall survival (OS) with comparison by log-rank test. Associations between potential prognostic variables and survival were estimated by Cox proportional hazard model. Other Statistical analyses were performed using SPSS package (Version 22, SPSS Inc., Chicago, IL, USA). All statistical tests were two-sided, with a *P*-value of less than 0.05 considered statistically significant.

## Results

### Overall trend of surgery in elderly patients

**Fig. 2:**
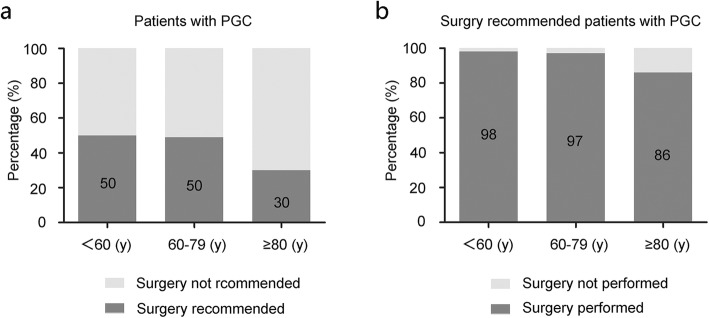
Proportion of surgery recommended or performed in different age groups. **a** Proportion of surgery recommended in different age groups of patients with proximal gastric carcinoma. **b** Proportion of surgery in different age groups of surgical candidates with proximal gastric carcinoma

A total of 59,698 patients with proximal gastric carcinoma identified from NCDB were initially screened into three age groups (< 60 yrs.: *n* = 16,766; 60–79 yrs.: *n* = 32,931; and ≥ 80 yrs.: *n* = 10,001). Among patients age ≥ 80 yrs., 2484 patients were recommended for surgery, with a significantly decreased proportion compared to the younger age groups (Fig. 2a, ≥ 80 yrs.: 30% vs. 60–79 yrs.: 50% vs. < 60 yrs.: 50%, *P* < 0.001). Among patients who were recommended for surgery, the proportion who ultimately underwent surgery decreased significantly in groups age ≥ 80 yrs. (86% vs. 97% for 60–79 yrs. vs. 98% < 60 yrs. groups, *P* < 0.001, Fig. 2b).

### Patient characteristics

A total of 2484 patients age ≥ 80 yrs. with resectable proximal gastric carcinoma identified from NCDB were eligible for the final analysis. Patients’ characteristics of the surgery group and no surgery group are summarized in Additional file 1: Table S1. Patients who underwent surgery were more likely to be younger, male gender, white race (*P* < 0.001). However, CDCI, tumor size, differentiation grade, and TNM stage did not significantly differ between the two groups. Patients who underwent surgery were less likely to receive chemotherapy (*P* < 0.001) or radiotherapy (*P* < 0.001). Detailed therapeutic strategies of the patients were summarized in Additional file 2: Table S2.

### Survival comparison between surgical and non-surgical groups (all recommended for surgery)

**Fig. 3:**
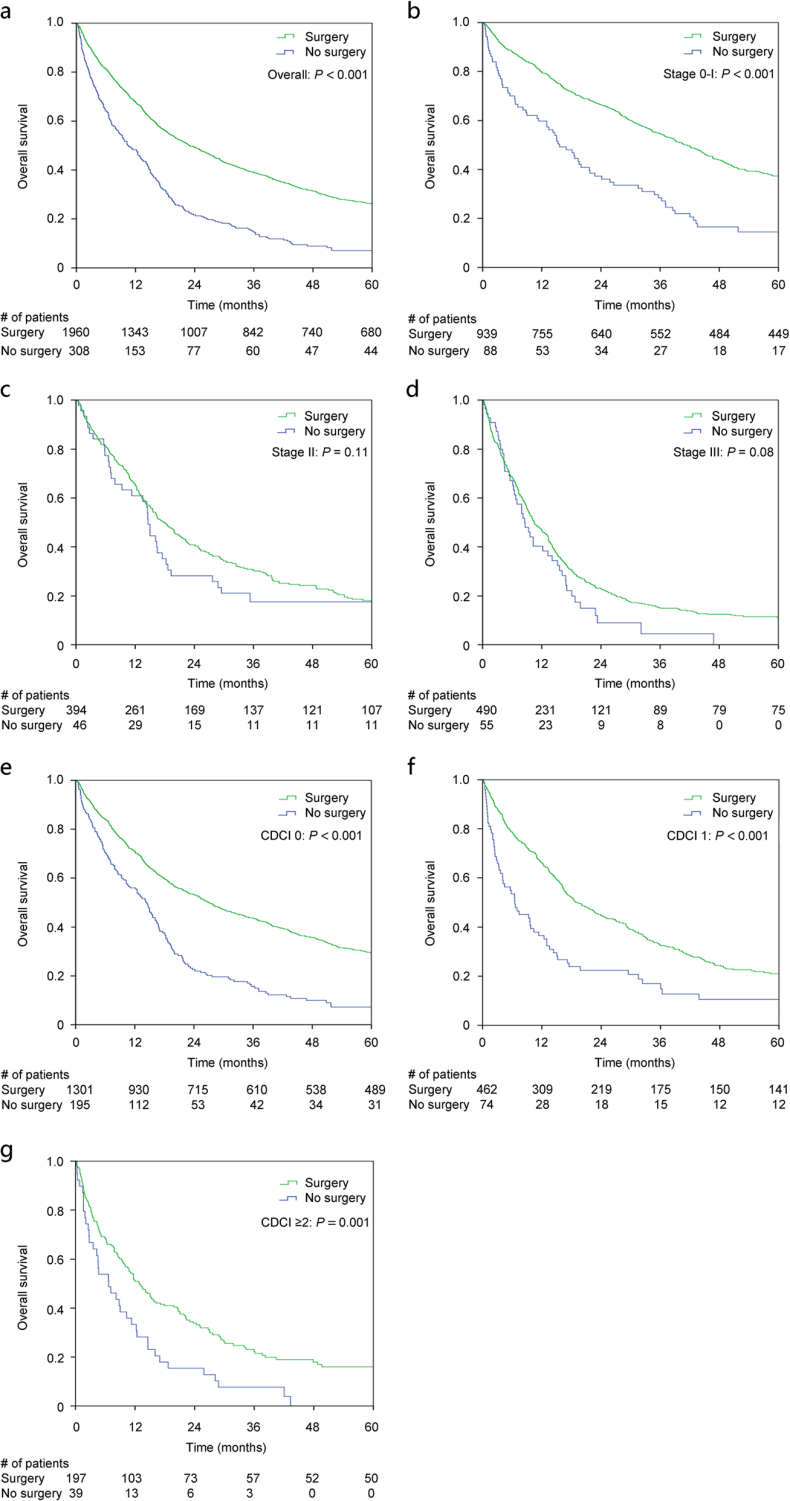
Kaplan-Meier survival curve of elderly patients who did or did not undergo surgery with resectable proximal gastric carcinoma from NCDB dataset. **a** All elderly patients with resectable proximal gastric carcinoma. **b** TNM stage 0 and I subgroup of patients; **c** TNM stage II subgroup of patients. **d** TNM stage III subgroup of patients; **e** CDCI score 0 subgroup of patients. **f** CDCI score 1 subgroup of patients. **g** CDCI score ≥ 2 subgroup of patients. CDCI: Charlson-Deyo Comorbidity Index

For patients who were recommended for surgery, there was no significant difference in CDCI, and TNM stage between surgical and non-surgical groups. It showed that these two group patients were comparable, and the selection bias was well controlled. Our data showed that patients who underwent surgery had a significantly better survival than those who did not undergo surgery (1-year OS: 68% vs. 48%; 3-year OS: 39% vs. 15%; 5-year OS: 26% vs. 7% respectively, *P* < 0.001, Fig. 3a), especially in stage 0-I patients (5-year OS: 37% vs. 14%, *P* < 0.001, Fig. 3b). No significant difference was observed in stage II (5-year OS: 18% vs. 18%, *P* = 0.11, Fig. 3c) and III patients (5-year OS: 11% vs. 0%, *P* = 0.08, Fig. 3d). A significant survival benefit was observed in both healthy patients (CDCI score = 0, 5-year OS: 29% vs. 7%, *P* < 0.001, Fig. 3e) and those with comorbidities (CDCI score = 1, 5-year OS: 21% vs. 11%, *P* < 0.001, Fig. 3f; and CDCI score ≥ 2, 5-year OS: 18% vs. 0%, *P* = 0.001, Fig. 3g). Interestingly, treatment with chemotherapy or radiotherapy did not significantly impact prognosis (HR: 0.90, 95% CI: 0.80–1.01, *P* = 0.08 for chemotherapy, and HR: 1.00, 95% CI: 0.88–1.13, *P* = 0.98 for radiotherapy). After adjustment for known factors including age, gender, CDCI, tumor size, differentiation grade, TNM stage using multivariable Cox proportional hazard model, surgery (HR: 0.66, 95% CI: 0.51–0.86, *P* = 0.002) remained a significant independent prognostic factor for elderly surgical candidates with resectable proximal gastric carcinoma (Table 1).

**Table 1:** Cox proportional hazards model for overall survival in the elderly patients with resectable proximal gastric carcinoma from NCDB database

Variables	Univariable Cox		Multivariable Cox	
	HR (95% CI)	*P*	HR (95% CI)	*P*
Age (per 1 SD)	1.05 (1.03–1.06)	<0.001	1.05 (1.03–1.07)	<0.001
Sex				
Female	Ref.			
Male	1.17 (1.06–1.29)	0.003	1.14 (1.00–1.29)	0.046
Race		0.20		
White	Ref.		–	
Black	1.05 (0.85–1.30)	0.63	–	
Asian	0.78 (0.58–1.04)	0.09	–	
CDCI Score		<0.001		<0.001
0	Ref.		Ref.	
1	1.28 (1.15–1.43)	<0.001	1.31 (1.14–1.51)	<0.001
≥ 2	1.77 (1.52–2.06)	<0.001	1.72 (1.41–2.08)	<0.001
Tumor size		<0.001		<0.001
≤ 2 cm	Ref.		Ref.	
2-4 cm	1.51 (1.31–1.75)	<0.001	1.23 (1.03–1.47)	0.02
4-6 cm	1.93 (1.65–2.26)	<0.001	1.34 (1.10–1.64)	0.004
>6 cm	2.19 (1.85–2.59)	<0.001	1.36 (1.10–1.68)	0.004
Differentiation grade		<0.001		0.005
Well	Ref.		Ref.	
Moderately	1.36 (1.11–1.67)	0.003	1.01 (0.78–1.31)	0.94
Poorly	1.94 (1.59–2.37)	<0.001	1.27 (0.98–1.65)	0.07
Undifferentiated	1.74 (1.19–2.56)	0.005	1.18 (0.74–1.87)	0.50
Analytic TNM stage		<0.001		<0.001
Stage 0-I	Ref.		Ref.	
Stage II	1.71 (1.50–1.94)	<0.001	1.36 (1.15–1.61)	<0.001
Stage III	2.62 (2.33–2.95)	<0.001	2.24 (1.89–2.65)	<0.001
If surgery				
No	Ref.		Ref.	
Yes	0.50 (0.44–0.57)	<0.001	0.66 (0.51–0.86)	0.002
If chemotherapy				
No	Ref.		–	
Yes	0.90 (0.80–1.01)	0.08	–	
If radiotherapy				
No	Ref.		–	
Yes	1.00 (0.88–1.13)	0.98	–	

*NCDB* National Cancer Database, *HR* Hazard ratio, *CI* Confidence interval, *CDCI* Charlson-Deyo Comorbidity Index. TNM was based on the T, N, and M elements defined by the American Joint Committee on Cancer (AJCC), the 7th edition. Analytic TNM Stage Group is assigned the value of reported Pathologic Stage Group. Clinical Stage Group is used if pathologic stage is not reported

### Survival analyses in patients who underwent surgery

Univariable Cox analyses in the subgroup who underwent surgery demonstrated that older age, male gender, higher CDCI, larger tumor size, lower differentiation grade, positive lymphovascular invasion, positive surgical margin, more number of lymph nodes (LNs) examined (continuous variable), and advanced TNM stage were associated with worse overall survival (Table 2). In addition, patients who underwent surgery with combined organ resection had a significantly worse survival (HR: 1.63, 95% CI: 1.33–2.00, *P* < 0.001), while those who underwent local excisions had a significantly better survival (HR: 0.61, 95% CI: 0.52–0.70, *P* < 0.001) when comparing with subtotal gastrectomy as reference. After adjustment using multivariable Cox regression, only age, CDCI, TNM stage, surgery type remained significant as independent factors for prognosis. Notably, neither chemotherapy (HR: 0.94, 95% CI: 0.82–1.08, *P* = 0.36), radiotherapy (HR: 0.97, 95% CI: 0.84–1.13, *P* = 0.72) nor the sequence of treatments (HR: 1.05, 95% CI: 0.77–1.43, *P* = 0.76) had an impact on survival in patients undergoing surgery (Table 2).

**Table 2:** Cox proportional hazards model for overall survival in elderly patients with resectable proximal gastric carcinoma who underwent surgery from NCDB database

Variables	Univariable Cox		Multivariable Cox	
	HR (95% CI)	*P*	HR (95% CI)	*P*
Age (per 1 SD)	1.04 (1.02–1.06)	<0.001	1.10 (1.05–1.15)	<0.001
Sex				
Female	Ref.		Ref.	
Male	1.21 (1.08–1.36)	0.001	1.03 (0.77–1.37)	0.87
Race		0.15		
White	Ref.		–	
Black	0.98 (0.76–1.26)	0.86	–	
Asian	0.70 (0.49–1.00)	0.05	–	
CDCI Score		<0.001		0.01
0	Ref.		Ref.	
1	1.32 (1.17–1.50)	<0.001	1.34 (0.99–1.81)	0.06
≥ 2	1.79 (1.50–2.12)	<0.001	1.72 (1.17–2.52)	0.006
Tumor size		<0.001		0.33
≤ 2 cm	Ref.		Ref.	
2-4 cm	1.56 (1.33–1.82)	<0.001	1.38 (0.91–2.09)	0.13
4-6 cm	2.03 (1.72–2.40)	<0.001	1.15 (0.71–1.85)	0.57
>6 cm	2.31 (1.94–2.76)	<0.001	1.05 (0.63–1.75)	0.86
Differentiation grade		<0.001		0.45
Well	Ref.		Ref.	
Moderately	1.24 (1.04–1.62)	0.02	1.01 (0.58–1.75)	0.98
Poorly	1.95 (1.57–2.42)	<0.001	1.25 (0.72–2.17)	0.42
Undifferentiated	1.72 (1.13–2.61)	0.01	0.92 (0.33–2.58)	0.88
Pathologic TNM stage		<0.001		<0.001
Stage 0-I	Ref.		Ref.	
Stage II	1.75 (1.52–2.01)	<0.001	1.41 (0.96–2.06)	0.08
Stage III	2.76 (2.43–3.14)	<0.001	3.61 (2.47–5.26)	<0.001
Lymphovascular invasion				
Negative	Ref.		Ref.	
Positive	1.75 (1.40–2.20)	<0.001	0.95 (0.69–1.32)	0.77
Type of surgery		<0.001		0.01
Subtotal gastrectomy	Ref.		Ref.	
Total gastrectomy	1.14 (0.97–1.32)	0.10	1.20 (0.85–1.69)	0.30
Gastrectomy with other organs	1.63 (1.33–2.00)	<0.001	1.88 (1.22–2.91)	0.004
Local excision	0.61 (0.52–0.70)	<0.001	0.64 (0.38–1.08)	0.10
Surgical margin				
Negative	Ref.		Ref.	
Positive	1.83 (1.57–2.12)	<0.001	1.68 (1.17–2.41)	0.01
Number of LNs examined (per 1 SD)	1.01 (1.01–1.02)	<0.001	0.99 (0.97–1.00)	0.10
Treatment facility		<0.001		0.14
AR-program	Ref.		Ref.	
INC-program	1.21 (1.01–1.45)	0.04	0.99 (0.61–1.60)	0.96
CCC-program	1.33 (1.18–1.49)	<0.001	1.36 (1.02–1.81)	0.03
CC-program	1.55 (1.25–1.92)	<0.001	1.36 (0.80–2.32)	0.26
If chemotherapy				
No	Ref.		–	
Yes	0.94 (0.82–1.08)	0.36	–	
If radiotherapy				
No	Ref.		–	
Yes	0.97 (0.84–1.13)	0.72	–	
Sequence of chemo/radiotherapy				
Upfront surgery	Ref.		–	
neoadjuvant therapy	1.05 (0.77–1.43)	0.76	–	

*NCDB* National Cancer Database, *LN* Lymph nodes, *HR* Hazard ratio, *CI* Confidence interval, *CDCI* Charlson-Deyo Comorbidity Index, *AR-program* Academic/Research Program, *INC-program* Integrated Network Cancer Program, *CCC-program* Comprehensive Community Cancer Program, *CC-program* Community Cancer Program. TNM was based on the T, N, and M elements defined by the American Joint Committee on Cancer (AJCC), the 7th edition

### Surgical risk and outcome related to facility

Nearly half of the elderly patients underwent surgery in academic/research program (AR-program, 992/2134, 46.5%). Compared to younger patients, 30-day and 90-day mortality rate was higher in patients age ≥ 80 yrs. (Additional file 3: Figure S1a, and S1b), however, the mortality rate was much lower for elderly patients who underwent surgery at academic and research (AR) program than that in integrated network cancer program, comprehensive community cancer program or community cancer program (30-day mortality: 1.5% in AR-program vs. 4.7, 3.6 and 6.6% in other three programs, *P* < 0.001; 90-day mortality: 6.2% in AR-program vs. 14.6, 13.6 and 16.4% in other three programs, *P* < 0.001) (Additional file 3: Figure S1c, and S1d). Consistent with the result of surgical risk, the survival outcome was also significantly better in patients underwent surgery in AR-program than those treated in integrated network cancer program, comprehensive community cancer program or community cancer program (5-year OS: 30% vs. 27% vs. 22% vs. 18% respectively, *P* < 0.001) (Table 2, and Additional file 4: Figure S2).

## Discussion

Gastric carcinoma in the elderly patients represents a distinct entity with specific clinicopathological characteristics and treatment response. Previous studies reported that elderly patients tend to have higher American Society of Anesthesiologists (ASA) physical status scores, more advanced stage, less resectability, as well as a poorer prognosis [11–13, 25]. On the other hand, proximal gastric carcinoma tends to be more common in elderly patients [12], and usually requires more complex and high risk procedures such as an esophagogastrectomy with esophagojejunostomy, or esophagogastrostomy. As a result, treatment strategies including surgical resection, chemotherapy, and radiation therapy are always controversial in elderly gastric carcinoma patients, especially for proximal tumors.

Most of previous studies reported similar risks and benefits of surgery for elderly GC patients when compared to their younger counterparts [25, 26], or reported comparable outcome between elderly GC patients who received surgery or not in all tumor locations [17]. However, no previous studies have focused on elderly proximal GC entity. Our study addresses this issue using the NCDB database.

We found that both the rate of surgery recommendation and the rate of surgery ultimately performed for elderly patients with proximal gastric carcinoma decreased dramatically (aged ≥80 yrs. vs. younger: 30% vs 50, and 86% vs 98%, respectively). This may be explained by that clinicians were reluctant to perform radical surgery for this group of patients due to comorbidities, high risk of perioperative morbidity and mortality, high proportion of late stage or metastasis, and short life expectancy [11]. Additionally, patients themselves may also contributed to this situation due to limited evidence of surgical benefit [27, 28].

More importantly, we found that within the group of elderly patients age ≥ 80 yrs., surgery could significantly improve OS, especially for early stage patients with resectable proximal gastric carcinoma. This finding is consistent with previous reports focusing on overall elderly patients with gastric cancer, regardless of tumor sites [17, 29, 30]. Moreover, the survival benefit of surgery was observed in both healthy and less healthy patients with certain comorbidities (CDCI ≥1), indicating that age-associated comorbidities should not be considered as absolute contraindication for surgery [13, 31, 32]. The gradually expanded indications for surgical treatment in elderly patients might attributed to the improvement of surgical techniques and postoperative intensive care treatments [33]. According to recent research, no significant differences in complications, morbidity, and hospital stay duration after surgery were found between younger patients and those older than 80 yrs. by using laparoscopy assisted gastrectomy [34]. Similar results were also reported that when surgery was performed safely, the survival rate of elderly patients was similar to that of the general population [26, 35, 36]. It is important to emphasize that our results are based on the patients who were deemed surgical candidates by treating clinicians. The treating clinicians play a pivotal role in assessing medical fitness, comorbidities, and the functional status of the elderly patient in order to determine the optimal treatment plan that will preserve the best possible quality and quantity of life [12].

Given the fear of the potential risks of surgery, it is generally claimed that elderly patients are often undertreated [37]. Although radical gastrectomy with D2 lymph node dissection has been widely accepted as the standard surgical approach for patient with gastric carcinoma, this aggressive approach has been questioned for elderly patients. While there are a limited number of studies reporting that higher lymph node examination could prolong survival without an increased postoperative mortality [38], most prior reports have demonstrated that extended lymph node dissection did not improve the 5-year OS of elderly patients and was associated with increased mortality and morbidity [17, 26, 39–41]. In our study, we found increased lymph node examination was a reverse prognostic factor, though it was not an independent risk factor in multivariable analyses. Moreover, patients undergoing extensive surgery with combined organ resection did not have an expected favorable survival outcome.

There are a few additional interesting findings from our study. We found that patients who were treated in academic or research program had a significantly lower 30-day mortality than a community cancer program. This might due to the surgical volume effect [42, 43] as shown in pancreatic surgery. The academic medical center usually has much more experience, comprehensive infrastructure and ready available services (intensive care unit, geriatric, cardiac, interventional radiology services) in taking care of complicated elderly population that usually has less physiological reserve.

In addition, while many randomized controlled trials (RCTs) have demonstrated that chemotherapy may improve 5-year OS for gastric carcinoma patients [44], patients age ≥ 80 yrs. were generally excluded or underrepresented by RCTs. As a result, the usefulness of applying chemotherapy or radiotherapy in elderly patients remains controversial. Our results also indicated that chemotherapy or radiotherapy had limited benefits in this elderly group, regardless if used in neoadjuvant or adjuvant setting if they received a curative surgical resection. This result was consistent with previous small cohort studies which demonstrated that elderly patients did not benefit from neoadjuvant or adjuvant treatment [45–48], especially for patients older than 80 years [49]. There are a limited number of studies that have reported a survival benefit for adjuvant chemoradiation therapy [50, 51]. The oncologic benefit of neoadjuvant or adjuvant therapy must be balanced with the potentially increased toxicities and decreased quality of life in elderly patients.

As this is a large population-based study, it has several potential limitations. First, given the retrospective design, all analyses are subject to selection biases and imbalances in unquantified variables. Second, this analysis is restricted to the evaluation of OS rather than disease-specific survival, and lacks relevant information such as the postoperative complications.

## Conclusions

Octogenarians with proximal gastric cancer appear to be undertreated in the US. Less-invasive approach (gastrectomy with less extensive lymph node dissection, and without joint organ resection) should be offered to patients who are considered potential surgical candidates in academic medical center, especially for those early stage patients. More evidence is needed to advocate or discourage the use of chemotherapy or radiotherapy in this group of patients.

## Supplementary information

**Additional file 1:** **Table S1.** Comparison of baseline variables between surgery and no surgery group in the elderly patients with resectable proximal GC from NCDB database.

**Additional file 2:** **Table S2.** Treatment strategy of elderly patients with resectable proximal GC from NCDB database.

**Additional file 3:** **Figure S1.** a-b: Postoperative 30-day and 90-day mortality in different age groups of patients with resectable proximal gastric carcinoma (PGC). c-d: Postoperative 30-day and 90-day mortality of elderly patients with resectable PGC treated in different facility.

**Additional file 4 **: **Figure S2.** Kaplan-Meier survival curve of elderly patients with resectable proximal gastric carcinoma treated in different facility.

## Data Availability

The data used in this study are available from National Cancer Database, which are used under license for the current study, and so are not publicly available. However, the statistic codes used during the current study are available from the corresponding author on reasonable request.

## Abbreviations

AR: Academic and research
CDCI: Charlson-Deyo Comorbidity Index
CI: Confidence interval
GC: Gastric cancer
GEJ: Gastroesophageal junction
HR: Hazard ratio
NCDB: National cancer database
OS: Overall survival
PGC: Proximal gastric cancer
