# Supplementary material for: Optimal treatment for elderly patients with resectable proximal gastric carcinoma: a real world study based on National Cancer Database
Source: BMC Cancer. 2019 Nov 9;19:1079. doi: 10.1186/s12885-019-6166-3 (PMC6842542; doi:10.1186/s12885-019-6166-3)
Supplement: Supplementary file 1 — Additional file 1: Table S1. Comparison of baseline variables between surgery and no surgery group in the elderly patients with resectable proximal GC from NCDB database. [file 12885_2019_6166_MOESM1_ESM.docx]

**Table S1.** Comparison of baseline variables between surgery and no surgery group in the elderly patients with resectable proximal GC from NCDB database

| **Variables** | **Elderly Patients with Resectable Proximal GC** | | | |
| --- | --- | --- | --- | --- |
|  | **Total (%)** | **Surgery (%)** | **No surgery (%)** | ***P#*** |
| **Age, year** Mean ± SD | 83.6 ± 3.0 | 83.4 ± 2.9 | 84.8 ± 3.5 | ＜0.001 |
| **Sex** |  |  |  | 0.002 |
| Male | 1626 (65.5) | 1422 (66.6) | 204 (58.3) |  |
| Female | 858 (34.5) | 712 (33.4) | 146 (41.7) |  |
| **Race** |  |  |  | ＜0.001 |
| White | 2243 (91.9) | 1950 (92.8) | 293 (86.2) |  |
| Black | 128 (5.2) | 92 (4.4) | 36 (10.6) |  |
| Asian | 69 (2.9) | 58 (2.8) | 11 (3.2) |  |
| **CDCI Score** |  |  |  | 0.15 |
| 0 | 1640 (66.0) | 1423 (66.7) | 217 (62.0) |  |
| 1 | 579 (23.3) | 492 (23.0) | 87 (24.9) |  |
| ≥2 | 265 (10.7) | 219 (10.3) | 46 (13.1) |  |
| **Tumor size** |  |  |  | 0.05 |
| ≤2cm | 574 (30.1) | 531 (30.3) | 43 (27.2) |  |
| 2-4cm | 613 (32.1) | 547 (31.3) | 66 (41.8) |  |
| 4-6cm | 412 (21.6) | 382 (21.8) | 30 (19.0) |  |
| ＞6cm | 309 (16.2) | 290 (16.6) | 19 (12.0) |  |
| **Differentiation grade** |  |  |  | 0.08 |
| Well | 195 (9.2) | 178 (9.7) | 17 (6.0) |  |
| Moderately | 869 (41.2) | 737 (40.3) | 132 (46.6) |  |
| Poorly | 998 (47.3) | 869 (47.6) | 129 (45.6) |  |
| Undifferentiated | 48 (2.3) | 43 (2.4) | 5 (1.8) |  |
| **Analytic TNM stage** |  |  |  | 0.34 |
| Stage 0-I | 1131 (51.3) | 1027 (51.8) | 104 (46.8) |  |
| Stage II | 482 (21.9) | 427 (21.6) | 55 (24.8) |  |
| Stage III | 591 (26.8) | 528 (26.6) | 63 (28.4) |  |
| **Chemotherapy** |  |  |  | ＜0.001 |
| No | 1992 (80.2) | 1736 (81.3) | 256 (73.1) |  |
| Yes | 492 (19.8) | 398 (18.7) | 94 (26.9) |  |
| **Radiotherapy** |  |  |  | ＜0.001 |
| No | 2056 (82.8) | 1816 (85.1) | 240 (68.6) |  |
| Yes | 428 (17.2) | 318 (14.9) | 110 (31.4) |  |

**Note.** Data were given as number (%) unless otherwise noted, missing data existed in different variables; GC: Gastric Carcinoma; NCDB: National Cancer Database; CDCI: Charlson-Deyo Comorbidity Index. TNM was based on the T, N, and M elements defined by the American Joint Committee on Cancer (AJCC), the 7^th^ edition.

Analytic TNM Stage Group is assigned the value of reported Pathologic Stage Group. Clinical Stage Group is used if pathologic stage is not reported. #: P value for χ2 test.
