# Supplementary material for: Optimal treatment for elderly patients with resectable proximal gastric carcinoma: a real world study based on National Cancer Database
Source: BMC Cancer. 2019 Nov 9;19:1079. doi: 10.1186/s12885-019-6166-3 (PMC6842542; doi:10.1186/s12885-019-6166-3)
Supplement: Supplementary file 2 — Additional file 2: Table S2. Treatment strategy of elderly patients with resectable proximal GC from NCDB database. [file 12885_2019_6166_MOESM2_ESM.docx]

**Table S2.** Treatment strategy of elderly patients with resectable proximal GC from NCDB database

| **Treatment strategy** | **Number of patients (%)** |
| --- | --- |
| **Surgery** | **2134 (100)** |
| Surgery alone | 1683 (78.9) |
| Surgery plus chemotherapy | 133 (6.2) |
| Neoadjuvant | 47 |
| Adjuvant | 67 |
| Unknown | 19 |
| Surgery plus radiotherapy | 53 (2.5) |
| Neoadjuvant | 2 |
| Adjuvant | 48 |
| Unknown | 3 |
| Surgery plus chemoradiotherapy | 265 (12.4) |
| Neoadjuvant | 124 |
| Adjuvant | 132 |
| Unknown | 9 |
| **No surgery** | **350 (100)** |
| Chemotherapy alone | 17 (4.9) |
| Radiotherapy alone | 33 (9.4) |
| Chemoradiotherapy alone | 77 (22.0) |
| No treatment | 223 (63.7) |
| **Note.** GC: Gastric carcinoma; NCDB: National Cancer Database. | |
