# Supplementary material for: Optimal treatment for elderly patients with resectable proximal gastric carcinoma: a real world study based on National Cancer Database
Source: BMC Cancer. 2019 Nov 9;19:1079. doi: 10.1186/s12885-019-6166-3 (PMC6842542; doi:10.1186/s12885-019-6166-3)
Supplement: Supplementary file 3 — Additional file 3: Figure S1. a-b: Postoperative 30-day and 90-day mortality in different age groups of patients with resectable proximal gastric carcinoma (PGC). c-d: Postoperative 30-day and 90-day mortality of elderly patients with resectable PGC treated in different facility. [file 12885_2019_6166_MOESM3_ESM.docx]

**Figure S1**


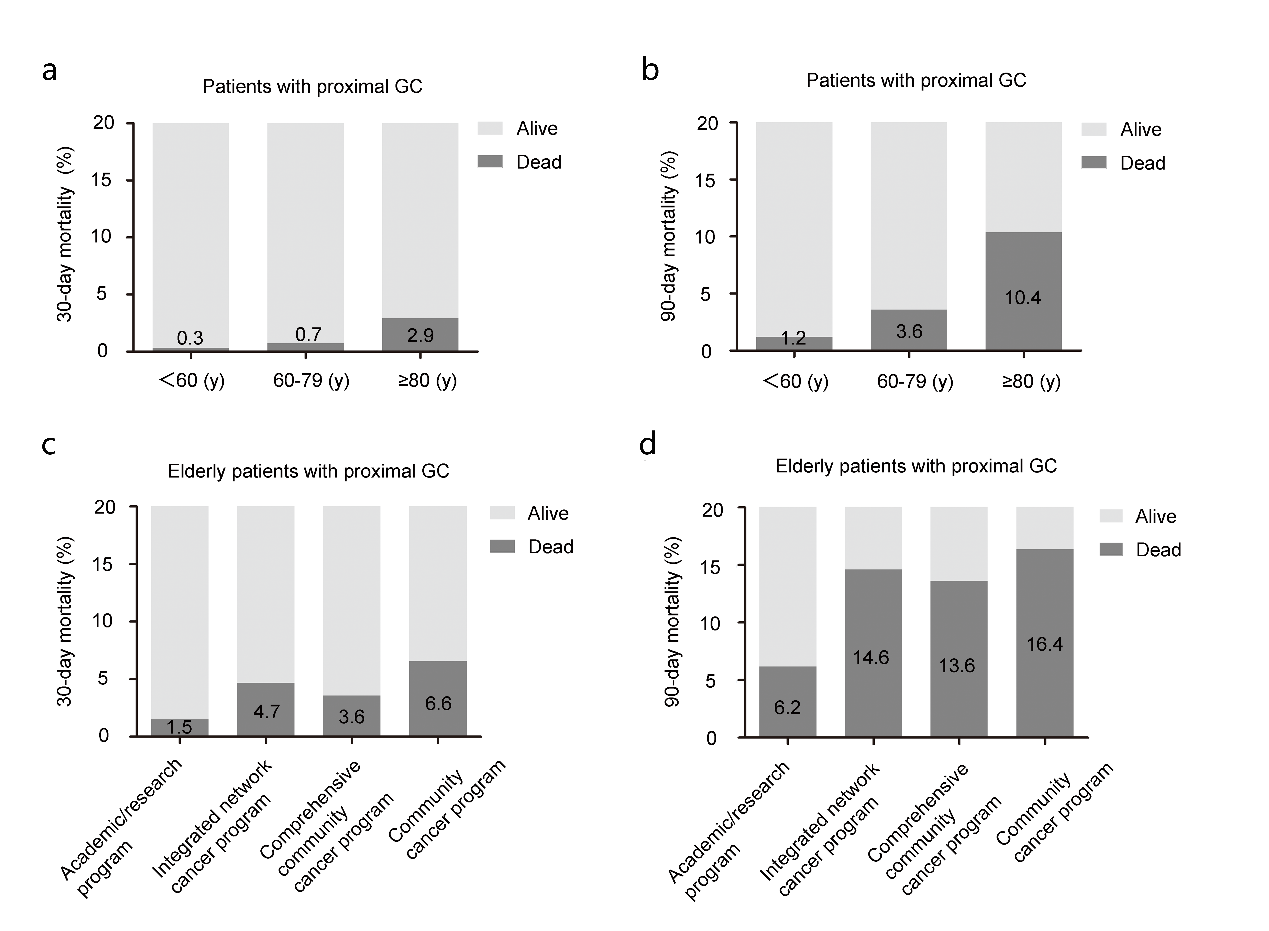


**Figure legends:** **a-b:** Postoperative 30-day and 90-day mortality in different age groups of patients with resectable proximal gastric carcinoma (PGC). **c-d:** Postoperative 30-day and 90-day mortality of elderly patients with resectable PGC treated in different facility.
