# Supplementary material for: Optimal treatment for elderly patients with resectable proximal gastric carcinoma: a real world study based on National Cancer Database
Source: BMC Cancer. 2019 Nov 9;19:1079. doi: 10.1186/s12885-019-6166-3 (PMC6842542; doi:10.1186/s12885-019-6166-3)
Supplement: Supplementary file 4 — Additional file 4 : Figure S2. Kaplan-Meier survival curve of elderly patients with resectable proximal gastric carcinoma treated in different facility. [file 12885_2019_6166_MOESM4_ESM.docx]

**Figure S2**

**
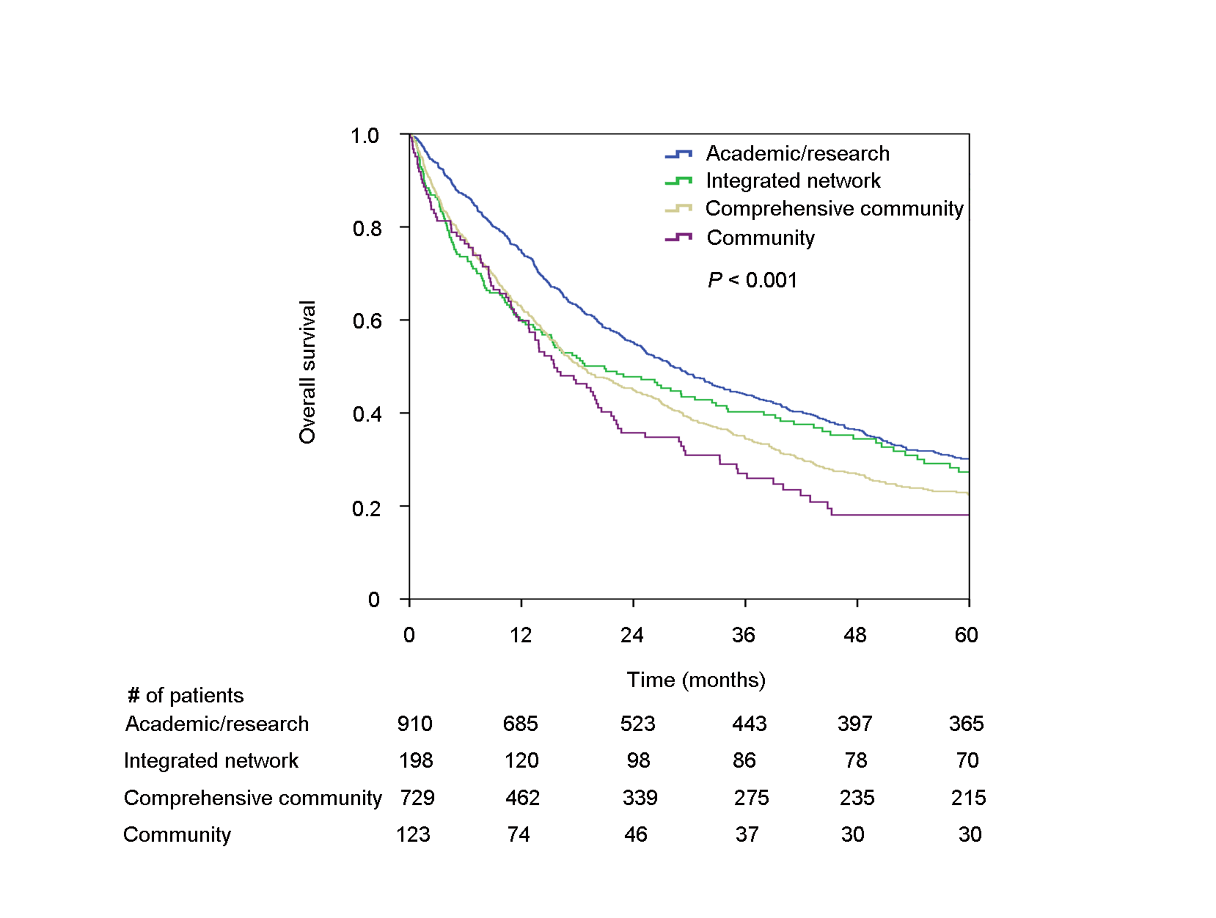
**

**Figure legends:** Kaplan-Meier survival curve of elderly patients with resectable proximal gastric carcinoma treated in different facility.
